# Cooperative Action of Oxidized Low-Density Lipoproteins and Neutrophils on Endothelial Inflammatory Responses Through Neutrophil Extracellular Trap Formation

**DOI:** 10.3389/fimmu.2019.01899

**Published:** 2019-08-09

**Authors:** Takashi Obama, Hitomi Ohinata, Takashi Takaki, Sanju Iwamoto, Naoko Sawada, Toshihiro Aiuchi, Rina Kato, Hiroyuki Itabe

**Affiliations:** ^1^Division of Biological Chemistry, Department of Pharmaceutical Sciences, Showa University School of Pharmacy, Tokyo, Japan; ^2^Division of Electron Microscopy, Showa University School of Medicine, Tokyo, Japan; ^3^Division of Physiology and Pathology, Department of Pharmacology, Toxicology and Therapeutics, Showa University School of Pharmacy, Tokyo, Japan

**Keywords:** neutrophil extracellular traps, oxidized low-density lipoprotein, endothelial cells, inflammatory response, HL-60 cell

## Abstract

The function of oxidatively modified low-density lipoprotein (oxLDL) in the progression of cardiovascular diseases has been extensively investigated and well-characterized with regards to the activation of multiple cellular responses in macrophages and endothelial cells. Although accumulated evidence has revealed the presence of neutrophils in vascular lesions, the effect of oxLDL on neutrophil function has not been properly investigated. In the present decade, neutrophil extracellular traps (NETs) gained immense attention not only as a primary response against pathogenic bacteria but also due to their pathological roles in tissue damage in various diseases, such as atherosclerosis and thrombosis. In this study, we investigated if oxLDL affects NET formation and if it is a risk factor for inflammatory reactions in endothelial cells. HL-60-derived neutrophils were stimulated with phorbol 12-myristate 13-acetate (PMA) for 30 min to induce NET formation, followed by incubation with 20 μg/mL native or oxidized LDL for additional 2 h. Culture media of the stimulated cells containing released NETs components were collected to evaluate NET formation by fluorometric quantitation of released DNA and detection of myeloperoxidase (MPO) by western blot analysis. NET formation of HL-60-derived neutrophils induced by PMA was significantly enhanced by additional incubation with oxLDL but not with native LDL. Treatment of HL-60-derived neutrophils with oxLDL alone in the absence of PMA did not induce NET formation. Furthermore, the culture media of HL-60-derived neutrophils after NET formation were then transferred to human aortic endothelial cell (HAECs) culture. Treatment of HAECs with the culture media containing NETs formed by HL-60-derived neutrophils increased the expression of metalloproteinase-1 protein in HAECs when HL-60-derived neutrophils were incubated with native LDL, and the expression was accelerated in the case of oxLDL. In addition, the culture media from NETs formed by HL-60-derived neutrophils caused the elongation of HAECs, which was immensely enhanced by coincubation with native LDL or oxLDL. These data suggest that oxLDL may act synergistically with neutrophils to form NETs and promote vascular endothelial inflammation.

## Introduction

The biological significance of oxidatively modified low-density lipoprotein (oxLDL) has been widely and extensively studied as a risk factor for the development and progression of cardiovascular diseases. Increase in oxLDL level is found not only in atherosclerotic lesions but also in the plasma and various organs (1, 2). The pathological process of vascular diseases induced by oxLDL has been explored in terms of the activation of multiple cellular responses in the macrophages and vascular endothelial cells. The underlying mechanisms of atherosclerotic plaque formation were strenuously analyzed via lipid accumulation in macrophage-derived foam cells and vascular endothelial cell dysfunction (3–5). It has also been demonstrated that neutrophils (polymorphonuclear leukocytes), the most abundant form of white cells, infiltrate into the atherosclerotic and thrombotic lesions (6, 7); however, the impact of neutrophils on the progression of atherosclerosis and its functional link with lipoproteins remains to be investigated.

Neutrophils have diverse functions, and they are crucial as the first line of defense responses against pathogenic bacteria due to their phagocytotic activity and ability to produce bactericidal reactive oxygen species (ROS). In 2004, Brinkmann et al. reported that neutrophil extracellular traps (NETs) cause mortality (8). NETs are evoked by pathological bacteria, lipopolysaccharide, cytokines, or other various stimuli that can induce decondensation of chromosome DNA via peptidyl arginine deiminase 4 (PAD4)-mediated histone citrullination, leading to the release of DNA, proteins, such as histones, myeloperoxidase (MPO), and elastase derived from nucleus and azurophil granules into the extracellular space. Signal transduction including ROS production by NADPH oxidase and mitochondria in neutrophils is involved in the process of NET formation (9). NETs have been studied with regards to the primary immune responses elicited against pathogenic bacteria via extracellular DNA trap. In the present decade, a growing evidence has revealed that NETs have pathophysiological functions in various diseases including rheumatoid arthritis (RA), systemic lupus erythematosus (10), cancer (11), Alzheimer's disease (12), metabolic diseases, and diabetes (13), indicating that NET formation may play a pivotal role in cellular dysfunction and histological damage leading to pathological conditions.

The presence of neutrophils in vasculature has been extensively investigated for cardiovascular diseases, such as abdominal aortic aneurysm (AAA) (14, 15), atherosclerosis, and thrombosis (6, 16, 17). Citrullinated histone known as a marker of NET formation is detected in the lesions of AAA (18, 19), thrombosis (20–22), atherosclerosis (23–25), and superficial erosion of atheroma linked with acute coronary syndrome (17). Several studies have demonstrated that citrullinated histone H4 and another NETs marker MPO-DNA complex in plasma are associated with the risk of cardiovascular diseases (26). The blockage of NET formation by Cl-amidine, a peptidyl arginine deiminase (PAD) inhibitor, reduced the atherosclerotic lesions in apoE-KO mice (27).

We have studied the pathological roles of oxLDL in vascular diseases (1, 2, 28, 29), and we investigated if oxLDL affects neutrophils in relation to vascular inflammation. It has already been established that both NETs and oxLDL independently act on the endothelial cells to evoke inflammatory responses and dysfunction; however, the role of native and modified lipoproteins in the progression of NET formation have remained unclear. In this study, we examined NET formation using HL-60-derived neutrophils in the presence of native LDL or oxLDL. Moreover, we assessed the possible synergistic effects of oxLDL treatment and NETs-forming cells on human aortic endothelial cells (HAECs).

## Materials and Methods

### Materials

All-*trans* retinoic acid (AtRA) and phorbol 12-myristate 13-acetate (PMA) were purchased from Wako Pure Chemical Industries, Ltd. (Osaka, Japan). Poly-L-lysine solution (P4707) and gelatin solution (G1393) were purchased from Sigma (Saint Louis, MO, USA). Anti-human MPO antibody (A0398) was purchased from Dako (Carpinteria, CA, USA). Anti-citrullinated histone H4 (citrulline 3) antibody (#07-596) and anti-histone H4 antibody (ab7311) were purchased from Merck KGaA (Darmstadt, Germany) and Abcam (Cambridge, UK), respectively. Horseradish peroxidase (HRP)-conjugated second antibody (anti-mouse IgG) (#3376) was purchased from Cell Signaling Technology, Inc. (Beverly, MA, USA). Anti VE-cadherin antibody (sc-9989), control mouse IgG (sc-2025), and control rabbit IgG (sc-2027) were purchased from Santa Crus Biotechnology, Inc. (Santa Cruz, CA, USA). Anti-matrix metalloproteinase-1 antibody (MMP-1; MAB901) was purchased from R&D Systems (Minneapolis, MN, USA). Anti-PKCβ antibody (GTX113252) was purchased from GeneTex Inc. (Irvine, CA, USA). SYTOX Green and second antibodies conjugated with Alexa Fluor 488 or 594 were purchased from Thermo Fisher Scientific (Rockford, IL, USA). Hoechst 33342 was purchased from Dojindo Laboratories (Kumamoto, Japan).

### Cell Culture

The human promyelocytic leukemia cell line HL-60 was purchased from American Type Culture Collection (ATCC), and the cells were cultured in RPMI-1640 medium (Wako Pure Chemical Industries, Ltd.) containing 5% fetal bovine serum (FBS) supplemented with 50 U/mL penicillin and 50 μg/mL streptomycin. HL-60 cells were incubated with 2 μM AtRA for 4 days to facilitate differentiation into mature neutrophils, as previously reported (30). Neutrophils were isolated from freshly drawn blood of healthy donors as previously reported (31). HAECs purchased from Lonza (Walkersville, MD, USA) were cultured in EGM-2 medium (Lonza) or Endothelial Cell Growth Medium 2 (PromoCell GmbH, Heidelberg, Germany). HAECs from passage 4–7 were used for this study. Morphological change in HAECs was evaluated by VE-cadherin staining. Circularity (4π × [Area]/[perimeter]^2^) of the cells was calculated using ImageJ software (NIH, USA) from at least 60 cells in each experimental condition.

### LDL Isolation and Oxidation

Human LDL was isolated from human plasma of healthy subjects, as described previously (32). Written informed consents in accordance with the Declaration of Helsinki were prepared and all human subjects voluntarily gave their signatures for participation in this study. The protocol was approved by the ethical committee of Showa University School of Pharmacy (No. 231). Briefly, the LDL fraction at a density of 1.019–1.063 was obtained via KBr density gradient ultracentrifugation, and was dialyzed against phosphate buffered saline (PBS) containing 250 μM EDTA in the dark to prevent divalent cation-mediated oxidation of LDL. Copper-mediated oxidation of LDL was performed by incubation of 0.2 mg/mL LDL with 5 μM CuSO_4_ at 37°C for 3 h. The reaction was stopped by cooling on ice and by addition of 250 μM EDTA.

### NETs Induction of HL-60-Derived Neutrophils or Human Neutrophils

HL-60-derived neutrophils or human neutrophils (1 × 10^5^ cells) were seeded on a poly-L-lysine-coated 12-well plate and were stimulated with 50 nM PMA for 30 min. After removing the culture medium, cells were washed once with the medium, followed by incubation in a medium containing 20 μg/mL native LDL or oxLDL for additional 2 h. The culture medium was collected and centrifuged at 700 × g for 3 min at room temperature (RT), and the supernatant was used for the quantitation of NETs and the stimulation of HAECs.

### Fluorometric Quantitation of NETs-DNA Release

Extracellular DNA released into the culture medium was quantified according to the previously reported protocol (33). Briefly, the culture medium collected as aforementioned was treated with 1 U/mL micrococcal nuclease (TAKARA Bio Inc. Kusatsu, Shiga, Japan) to partially digest NETs-derived DNA, and was centrifuged at 1,800 × g for 10 min. The DNA fragments recovered in the supernatant were quantified using 1 μM SYTOX Green and a fluorometer (VARIOSCAN, Thermo Fisher).

### Visualization of NETs

HL-60-derived neutrophils were seeded on poly-L-lysine-coated chamber slide (Thermo Fisher) and were stimulated as aforementioned. Cells were treated with 1 μg/mL Hoechst 33342 and 5 μM SYTOX green for 15 min, fixed with 4% paraformaldehyde in PBS for 15 min, and then permeabilized with 0.5% Triton X-100 in PBS for 1 min. After blocking cells with 5% BSA in PBS, they were incubated with the primary antibody against citrullinated histone H4 at RT for 1 h. After further washing with PBS, the cells were incubated with Alexa Fluor 594-conjugated anti-rabbit IgG antibody at RT for 45 min. Following this, the cells were washed with PBS, mounted with SlowFade™ Diamond (Thermo Fisher Scientific), and observed using a fluorescence microscope (BZ-9000, KEYENCE, Osaka, Japan).

Moreover, the chamber slide was used in an electron microscope for the analysis of NET formation of HL-60-derived neutrophils. The cells fixed with 4% paraformaldehyde were treated with 0.5% OsO_4_ for 30 min and were dehydrated by immersion in increasing concentrations of ethanol solutions (50, 60, 70, 80, 90, and 100%). The samples were dried using critical point dryer (HCP-2, HITACHI), and the surfaces of the samples were coated with 5 nm platinum layer using an auto fine coater (JEC-3000FC, HITACHI), followed by observation under a scanning electron microscope (SEM) (FlexSEM 1000, Hitachi High-Technologies Co., Tokyo, Japan).

### Culture and Stimulation of HAECs

HAECs were cultured on 0.1% gelatin-coated 6-well plates until confluent. The cells were washed with fresh medium and cultured for 30 min. Culture medium of HL-60-derived neutrophils after NET formation (333 μL) was added to 1 mL of HAECs culture medium, and the cells were incubated for 24 h. Notably, the final concentration of lipoproteins was 5 μg/mL, and the HAECs were lysed in SDS-PAGE sample buffer.

### Immunoblot Analysis

Cell lysates or culture medium was subjected to 15% gel SDS-PAGE or gradient gel native-PAGE, respectively, and then transferred to polyvinylidene difluoride (PVDF) membranes. After blocking the membrane with 2% skim milk (Wako Pure Chemical Industries, Ltd., Osaka, Japan) in Tris-buffered saline (TBS) containing 0.1% Tween-20 (TTBS) for 1 h, the membranes were treated with a primary antibody in the blocking solution at 4°C overnight. Furthermore, after washing the membrane with TTBS, the membrane was treated with a secondary antibody in TTBS containing 0.5% skim milk at RT for 2 h. The protein bands were detected using ECL-plus western blotting detection reagent (GE Healthcare UK Ltd., Buckinghamshire, UK) by ImageQuant LAS500 (GE Healthcare). Densitometric analysis was performed using ImageJ software.

### Immunocytochemistry

HAECs were cultured on 0.1% gelatin-coated glass coverslip (Matsunami Glass Inc., Ltd., Osaka, Japan) placed on a 12-well plate. After stimulation, the cells were fixed with 4% paraformaldehyde in PBS for 15 min, permeabilized with 0.5% Triton X-100 in PBS for 1 min, and blocked with 5% BSA in PBS. The cells were then washed with PBS and incubated with the primary antibody against VE-cadherin (with shaking) at RT for 1 h. After washing with PBS, the cells were incubated with Alexa Fluor 488-conjugated anti-mouse IgG antibody (ThermoFisher Scientific) at RT for 45 min. After further washing with PBS, the cells were mounted with SlowFade™ Diamond using 4′,6-diamidino-2-phenylindole, dihydrochloride (DAPI; ThermoFisher Scientific), and observed under a fluorescence microscope.

### Statistical Analysis

Data are presented as the mean ± standard deviation. The results were analyzed using Tukey–Kramer *post-hoc* test. Statistically significant difference was evaluated at *p* < 0.05.

## Results

### OxLDL Enhances NET Formation of HL-60-Derived Neutrophils and Human Peripheral Blood Neutrophils Induced by PMA

**Figure 1:**
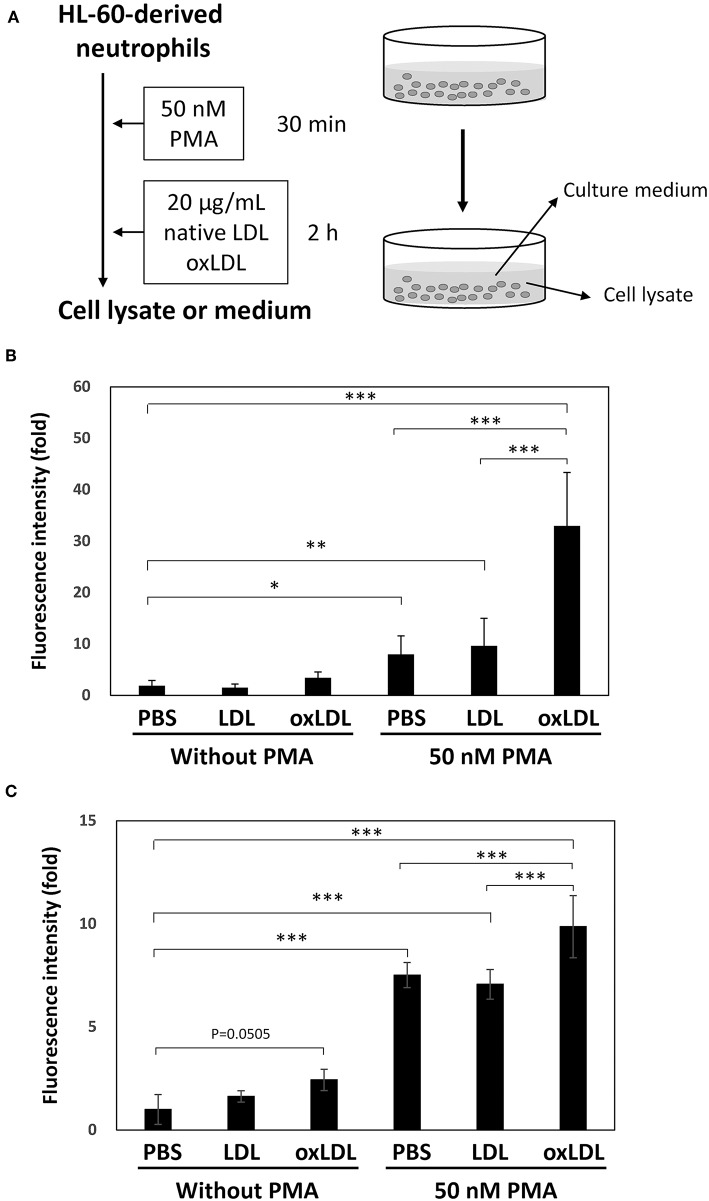
NET formation was enhanced by oxLDL treatment after priming HL-60-derived neutrophils with PMA. **(A)** The experimental procedure is illustrated. HL-60 cells were stimulated with 2 μM all-trans retinoic acid for 4 days to differentiate into neutrophils. The cells were stimulated with 50 nM PMA for 30 min and then treated with 20 μg/mL native LDL or oxLDL for additional 2 h. **(B)** Fluorometric quantification of DNA release from HL-60-derived neutrophils and **(C)** Human peripheral blood neutrophils. Collected culture medium was treated with micrococcal nuclease and then stained with 1 μM SYTOX Green. The data are presented as the mean ± SD of four independent experiments. Each experiment contains triplicate wells. Asterisks indicate statistical significance. ^*^p < 0.05, ^**^p < 0.005, ^***^p < 0.001.

Extracellular DNA release from HL-60-derived neutrophils under PMA treatment and treatments with native LDL or oxLDL (Figure 1A) was determined by fluorometric quantitation of DNA in the culture medium with SYTOX Green. DNA release was significantly induced by PMA treatment. The DNA release increased 3-fold by additional oxLDL treatment, but not by native LDL (Figure 1B). A similar response was observed when human peripheral blood neutrophils were assessed under the same experimental conditions (Figure 1C).

**Figure 2:**
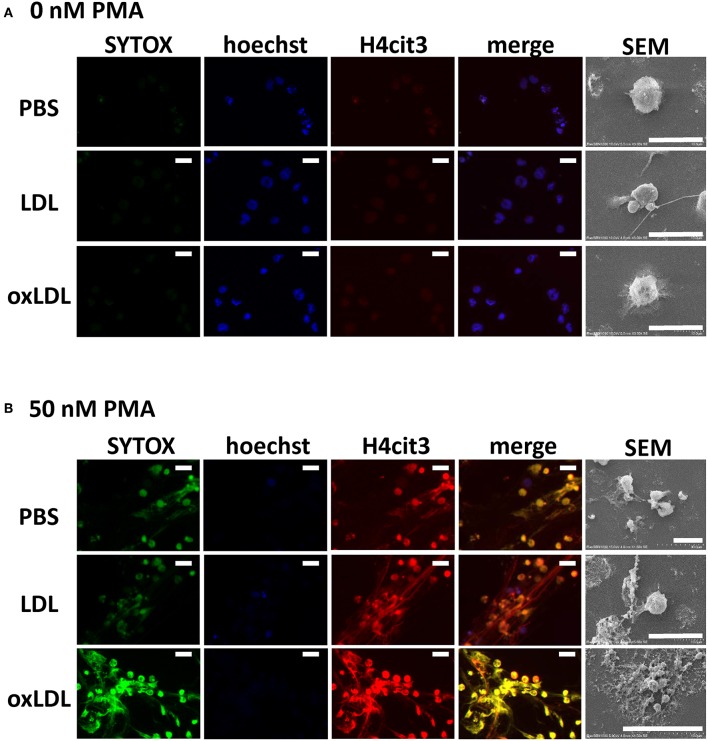
Microscopic observation of NET formation of HL-60-derived neutrophils. Cells were stimulated **(A)** without or **(B)** with 50 nM PMA for 30 min, followed by 20 μg/mL native LDL or oxLDL for additional 2 h. Cells were fixed and stained with Hoechst 33342 (blue), SYTOX Green (green), and citrullinated histone H4 (red), and then visualized by fluorescence microscopy and scanning electron microscopy (SEM). The results are representative of more than three experiments. Scale bars indicate 20 μm.

DNA release from the cells and histone citrullination in NETs structure were examined by immunofluorescent microscopy. Extracellular DNA and citrullinated histone H4 were microscopically visualized using SYTOX Green and anti-histone H4 (citrulline 3) antibody, respectively. When HL-60-derived neutrophils were incubated without PMA, slight staining of SYTOX Green and citrullinated histone H4 was observed (Figure 2A). When HL-60-derived neutrophils were stimulated with PMA, citrullinated histone H4 generated by the cells colocalized with extracellular DNA stained by SYTOX Green (Figure 2B). Neither native LDL nor oxLDL in the absence of PMA was sufficient for the induction of histone citrullination as well as DNA release, and this was demonstrated by the Hoechst 33342 staining of nuclei (Figure 2A). Strong overlapping signals of released DNA and citrullinated histones were detected when the neutrophils were treated with PMA followed by oxLDL. In similar conditions, loss of cell nuclei, released strings, and cellular debris were observed with SEM (Figure 2B).

**Figure 3:**
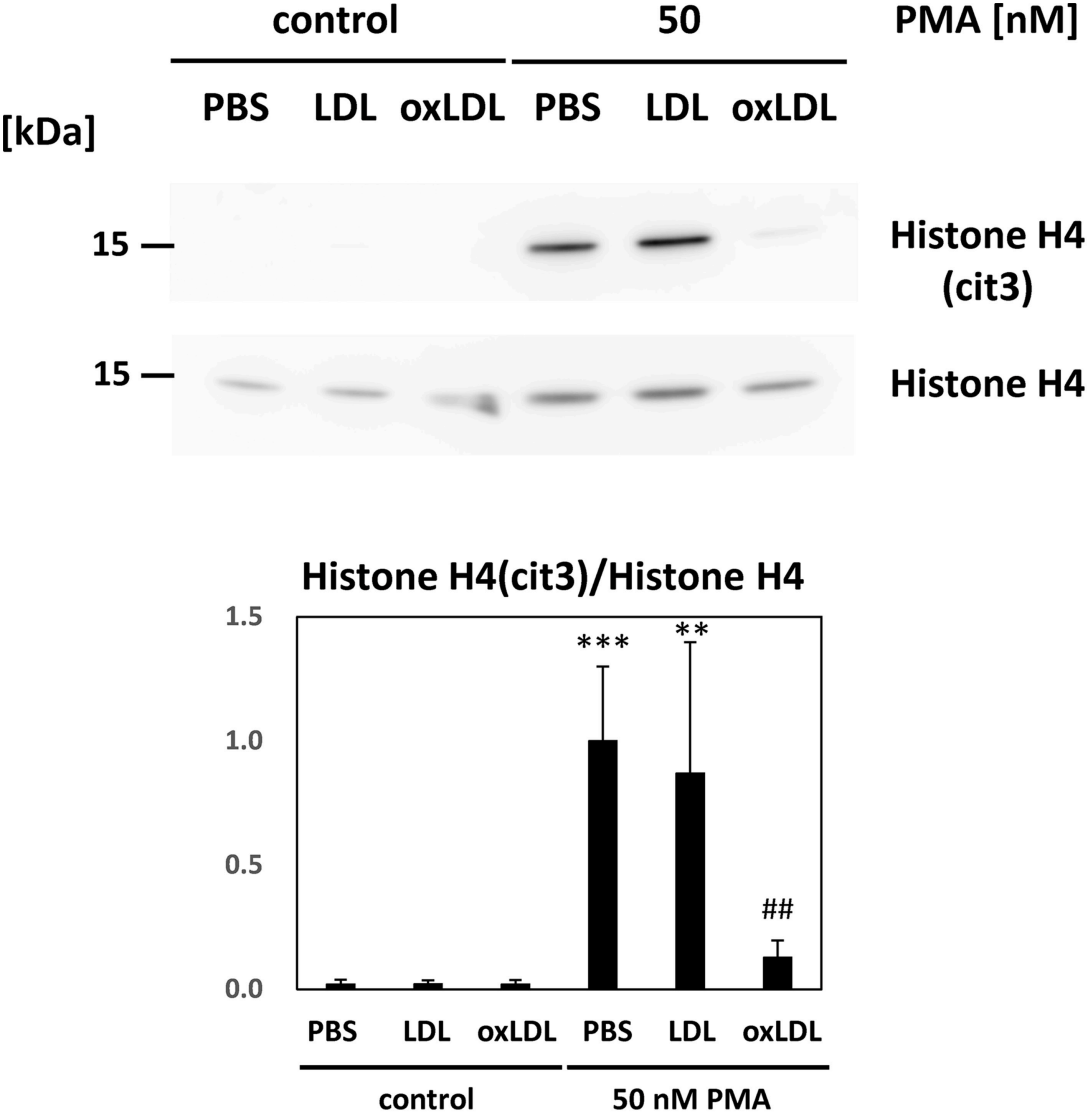
Citrullination of histone H4 in the HL-60-derived neutrophils was evaluated. Cells were stimulated with 50 nM PMA for 30 min and followed by 20 μg/mL native LDL or oxLDL for additional 2 h. Citrullinated histone H4 in the cell lysate of HL-60-derived neutrophils was analyzed by western blot analysis. Each band was normalized by histone H4 of the corresponding sample. Quantitative data presented in the bar graph are the mean ± SD of three experiments. Asterisks indicate statistical significance against the condition “PBS without PMA”; ^**^p < 0.005, ^***^p < 0.001. Sharps indicate statistical significance against the condition “PBS with PMA”; ^##^*p* < 0.005.

Immunoblot analysis of HL-60-derived neutrophils was carried out to determine histone citrullination during NET formation (Figure 3). Citrullinated histone H4 was formed under PMA stimulation. The level of citrullinated histone in the cell lysate decreased with additional oxLDL treatment. Native LDL or oxLDL treatment alone did not cause histone citrullination in HL-60-derived neutrophils, indicating that the lipoproteins did not activate PAD4 in the cells to initiate histone citrullination.

These data suggest that extracellular release of DNA into the culture medium is induced by PMA and subsequent oxLDL treatment significantly enhanced the release of DNA and associating proteins.

### OxLDL Treatment Following NET Formation Increased MPO Release From HL-60-Derived Neutrophils

**Figure 4:**
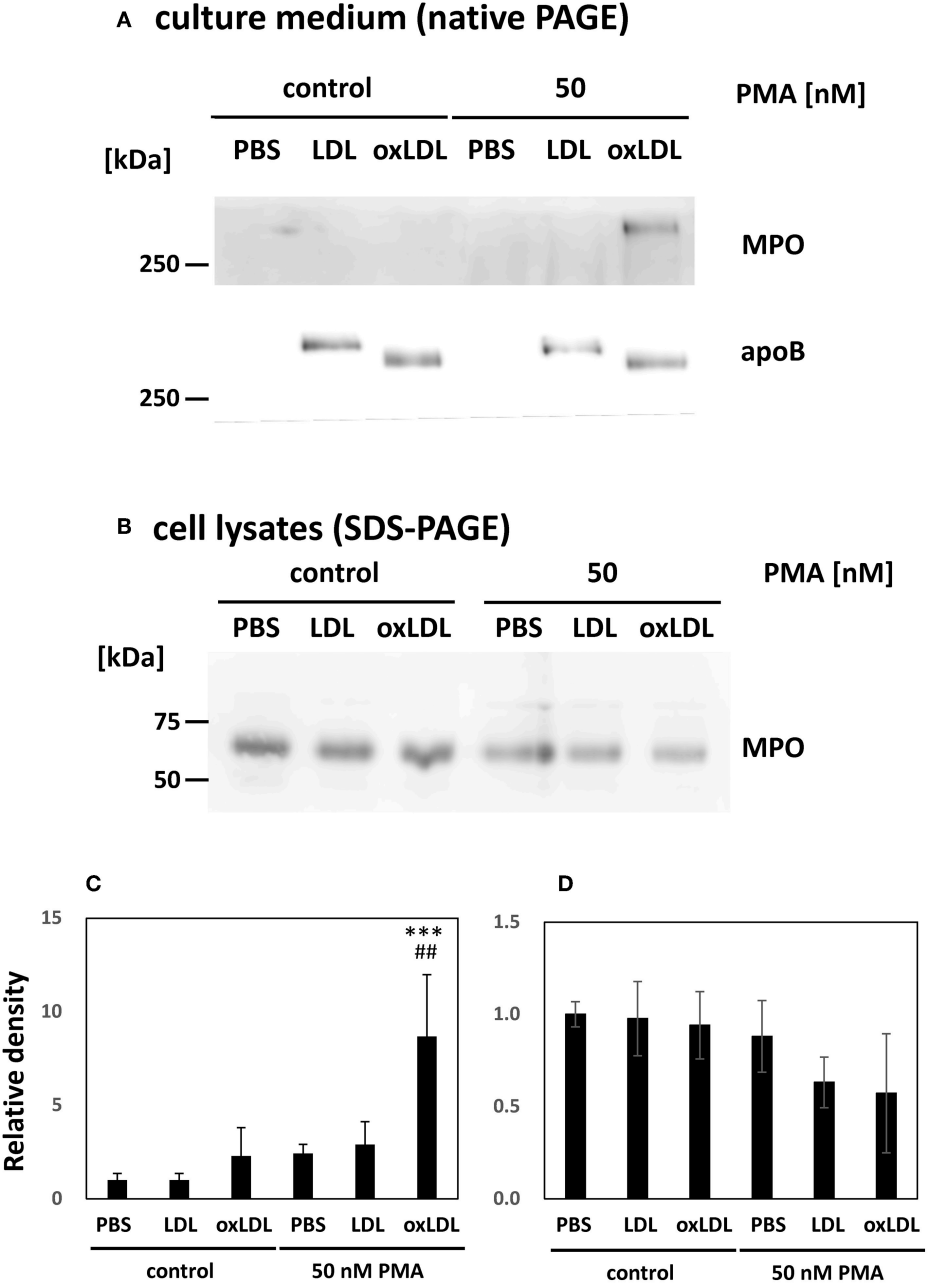
Myeloperoxidase (MPO) released from PMA-stimulated neutrophils was enhanced by oxLDL treatment. HL-60-derived neutrophils were stimulated with 50 nM PMA for 30 min following 20 μg/mL native LDL or oxLDL for additional 2 h. **(A,C)** Western blot analysis of culture media of the cells to detect MPO and apolipoprotein B-100 (apoB) following native PAGE on a 3–20% gradient gel. **(B,D)** Western blot analysis of MPO in cell lysates of HL-60-derived neutrophils following 12% gel SDS-PAGE. Quantitative data presented in the bar graph are the mean ± SD of three experiments. Asterisks indicate statistical significance against the condition “PBS without PMA”; ^***^p < 0.001. Sharps indicate statistical significance against the condition “PBS with PMA”; ^##^*p* < 0.005.

Western blot analysis of the culture media was carried out to detect MPO. MPO is a heterotetrametric protein containing 60 kDa subunit, which can be recognized by the antibody used (34). MPO released from HL-60-derived neutrophils was not clearly visible in the absence of PMA stimulation. Notably, MPO release increased dramatically when the cells were stimulated with PMA and subsequently treated with oxLDL, whereas native LDL treatment of stimulated cells did not induce MPO release (Figures 4A,C). Intracellular MPO decreased by 40% after the stimulation of the cells with PMA and oxLDL (Figures 4B,D). MPO in the cell lysate was detected at 60 kDa as presented in Figures 4B,D; however, the band for extracellular MPO appeared larger than 250 kDa on a native PAGE (Figures 4A,C). The high molecular weight MPO band colocalized with apoB-100, a major protein component of LDL, on native PAGE, suggesting that released MPO interacts with oxLDL. Apparent size of the MPO band was not affected by treating the culture media with DNase I to cleave NETs DNA prior to native-PAGE analysis (Supplemental Figure 1). These data indicate that MPO released into the culture medium interacts with lipoproteins.

### NETs Components Induced Inflammatory Responses in HAECs

**Figure 5:**
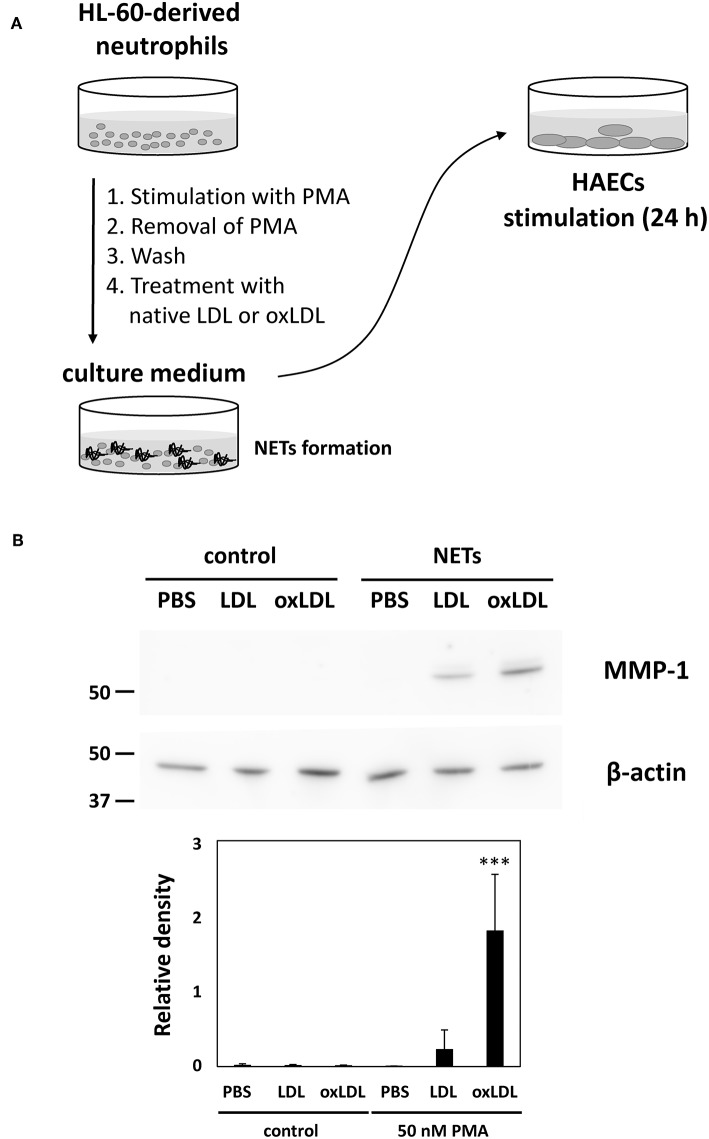
Enhanced NETs release from HL-60-derived neutrophils by oxLDL treatment is associated with increased inflammatory responses in HAECs. **(A)** Experimental procedure for the activation of HAECs by NETs components. HL-60-derived neutrophils were stimulated with 50 nM PMA for 30 min. The culture supernatant was discarded, and the cells were washed with fresh medium to remove PMA, then the cells were subjected to subsequent treatment with 20 μg/mL LDL or oxLDL for 2 h. The culture medium of the HL-60-derived neutrophils (333 μL) was transferred to 1 mL of HAECs culture medium (final concentration of 5 μg/mL lipoproteins) and cultured for 24 h. **(B)** Expression of MMP-1 in HAECs was analyzed by using western blot. The intensity of each band was normalized by the corresponding band of β-actin. Quantitative data presented in the bar graph are the mean ± SD of three experiments. Asterisks indicate statistical significance against the condition PBS without PMA; ^***^p < 0.001.

To further investigate the possible effects of enhanced NET formation by oxLDL treatment, the culture media of HL-60-derived neutrophils were transferred to HAECs and incubated for 24 h. In this series of experiments, HL-60-derived neutrophils were treated with native LDL or oxLDL after washing the cells in order to remove PMA. Subsequently, HAECs were exposed to NETs components released from PMA-stimulated HL-60-derived neutrophils, with 5 μg/mL lipoproteins (Figure 5A). Induction of MMP-1 protein expression in HAECs was observed after treatment with NETs components collectively with native LDL or oxLDL, whereas MMP-1 was not induced without NETs components even though HL-60-derived neutrophils were treated with native LDL or oxLDL (Figure 5B). The response of HAECs is presumably parallel to the release of NETs components from HL-60-derived neutrophils. Although PMA is also known as a powerful inducer of PKC expression in HAECs (35, 36), culture medium of NET-induced HL-60-derived neutrophils did not induce PKCβ protein expression in HAECs under our experimental conditions (Supplemental Figure 2), indicating that the transfer of PMA from culture medium of PMA-treated HL-60-derived neutrophils could be ignored.

### Morphological Changes in HAECs Stimulated With Soluble NETs Components

**Figure 6:**
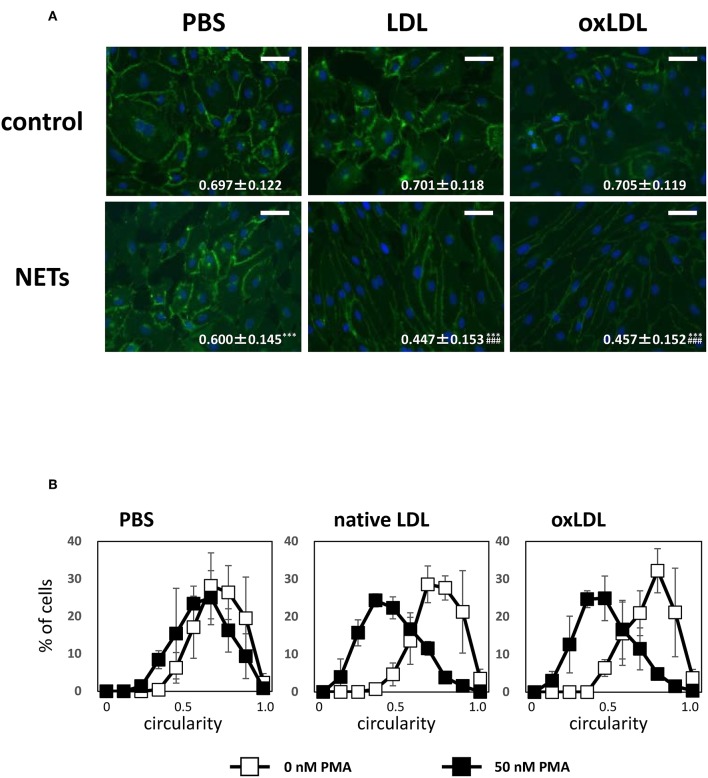
NETs components induce morphological alteration of HAECs, which is enhanced by coincubation with LDL or oxLDL. HAECs were stimulated as indicated in the figure legends of Figure 5. **(A)** Changes in the cell shapes of HAECs were evaluated by VE-cadherin (green) staining. Scale bars indicate 100 μm. Circularity of each cells was calculated using Image J software. Average circularity in each experimental condition is presented at the bottom of the images as the mean ± SD of at least 60 cells. Asterisks indicate statistical significance against each condition without PMA; ^***^p < 0.001. Sharps indicate statistical significance against PBS with PMA; ^###^*p* < 0.001. **(B)** Distribution of circularity of each HAECs is shown in white (control) and black (NETs) symbols. The data presented are the mean ± SD of three independent experiments.

Moreover, the morphological changes in HAECs were observed by carrying out immunostaining with VE-cadherin. Treatment with culture media from NETs-induced HL-60-derived neutrophils caused the elongation of HAECs which reduced cell circularity. The average circularity of HAECs was significantly decreased from 0.697 ± 0.122 to 0.600 ± 0.145 when the culture medium of HL-60-derived neutrophils stimulated with PMA was added to HAECs. Such effects on the cell shape of HAECs were immensely enhanced when HL-60-derived neutrophils were additionally treated with native LDL (from 0.701 ± 0.118 to 0.447 ± 0.153) or oxLDL (from 0.705 ± 0.119 to 0.457 ± 0.152; Figure 6B). In addition, decrease in HAEC circularity was associated with reduced fluorescence intensity of VE-cadherin-stained HAECs (Figure 6A). Notably, such changes were not observed in the cell shape of HAECs when they were incubated with native LDL or oxLDL alone at the same concentration (5 μg/mL as the final concentration). These data indicate that bioactive components generated during NET formation may act on HAECs, and the activity was greatly facilitated in the presence of native LDL and oxLDL. After incubating HAECs for 24 h, colocalization of MPO with apoB of native LDL increased (Supplemental Figure 3), indicating that the oxidative modification of native LDL may proceed to increase its electronegativity.

## Discussion

In this study we demonstrated that oxLDL significantly augmented NET formation by HL-60-derived neutrophils and human peripheral blood neutrophils, as well as NETs-dependent inflammatory activation in vascular endothelial cells. Neutrophil is one of the major cell types recruited in the lesions of vascular diseases. OxLDL accumulates in the atherosclerotic lesions and exhibits various biological activities against vascular cells to promote inflammatory responses and dysfunctions; however, the possible effect of oxLDL on neutrophils with regards to the promotion of vascular diseases has not been studied. To the best of our knowledge, this study was the first to investigate the cooperative effect of neutrophils and oxLDL on vascular cells.

NETs were initially reported to act as one of the natural immune responses to bacterial infection by releasing their own chromosomal DNA collectively with the nuclear, cytoplasmic, and granular proteins; however, excess NET formation has been implicated in pathophysiological disruption in various organs. Neutrophil infiltration is detected in the intima of atherosclerotic lesions in human vessels, specifically in acute coronary syndrome (6), myocardial infarction, and thrombotic lesions (7). Certain studies demonstrated that NET formation is involved in the activation and dysfunction of endothelial cells (37), promotion of angiogenesis (38), and progression of atherosclerotic lesions and cardiovascular diseases (16). Obesity-induced endothelial dysfunction was prevented by NETs inhibition (39). These studies would provide a promising new approach for the treatment of vascular diseases. Considering such information on the potential importance of NETs in vascular diseases and the presence of neutrophils in atherosclerotic lesions, we hypothesized that oxLDL is involved in NET formation.

Quantitative evaluation of NET formation is difficult; however, we introduced a fluorometric quantitation procedure to determine the extracellular DNA. Based on fluorometric quantitation of released NETs-DNA and western blot of citrullinated histone H4 in cell lysates, PMA-induced NET formation of HL-60-derived neutrophils was significantly accelerated by oxLDL at a concentration of 20 μg/mL, which was insufficient for NETs induction without PMA stimulation. In addition, the immunofluorescent and electron microscopic analyses of released DNA confirmed the drastic change in the cells. In a previous study, Awasthi et al. reported that oxLDL was able to induce NET-like structure against neutrophils; however, they treated neutrophils with 100 μg/mL oxLDL, whereas histone citrullination was not assessed (40). In the previous study, the circulating oxLDL level in human plasma was estimated to be 7–35 μg/mL (41, 42). In our present study, we utilized 20 μg/mL oxLDL at physiologically relevant concentration and confirmed that histone citrullination was not induced by stimulation with oxLDL alone. Thus, the effect of oxLDL on HL-60-derived neutrophils represents the enhancement of NET formation. According to a previous report, the blocking of toll like receptor-2 and -6 partially reduced oxLDL-induced NET formation (40), and these receptors may recognize and uptake oxLDL to induce enhancement of NET formation.

Accelerated NET formation induced by subsequent treatment with oxLDL was associated with enhanced release of MPO into the culture media. Native-PAGE analysis demonstrated that extracellular MPO interacted with apoB, corresponding with the results of previous studies which revealed that MPO binds to LDL and oxLDL (43, 44). MPO may contribute to high electronegative affinity with oxLDL because MPO is a highly cationic protein (45). MPO mediates the oxidative modification on the surface of LDL via ROS formation and/or MPO-catalyzed nitration and chlorination of proteins (46). Presumably, increased NET formation by oxLDL also promotes oxLDL production. Elevated plasma MPO levels were associated with acute myocardial infarction (47). In accordance, elevated level of MPO-DNA complex, the marker of NETosis, correlated with the severity of coronary artery diseases (26). A significant positive correlation between oxLDL and MPO in plasma from patients with ST-elevation myocardial infarction has been reported (7). MPO mediates the conversion of hydrogen peroxide to hypochlorous acid (HOCl)—a compound which induces the production of chlorinated lipid. Recently, 2-chlorofatty acid was reported as one of the lipid mediators that induce NET formation (48). MPO has also been implicated in endothelial dysfunction (49) and atherosclerosis (50). Our observation and other reports collectively suggest a possibility that circulating oxLDL acts as a carrier of MPO derived from neutrophils and contributes to the development of vascular inflammation.

As many studies, including ours (51), have previously reported, oxLDL contains various lipids including oxidized phosphatidylcholines, lysoPCs, and fatty acids. Neutrophils are susceptible to the toxic action of free fatty acids which causes cell death accompanied by direct membrane perturbation (52). Recent studies have extensively reported that lipid mediators are involved not only in NETosis but also in other types of programmed neutrophil death. Oxidized phospholipids and lysoPCs are mediators of NET formation (40). Ceramides are involved in NET induction (19, 53). Some studies demonstrated that various sphingolipid signaling mediated by sphingolipids and their analogs play roles in programmed neutrophil death, such as apoptosis and necroptosis (54, 55). Lipidomic analysis would be necessary to investigate the mechanism underlying oxLDL-mediated enhancement of NET formation.

Our data demonstrate that the culture medium of HL-60-derived neutrophils, which was treated with PMA and subsequently with oxLDL induced marked alteration of HAECs. Under suitable conditions, MMP-1 protein expression was induced in HAECs. MMP-1, also known as interstitial collagenase, is involved in the degradation of vascular extracellular matrix including collagen type I, II, and III. Elevated level of MMP-1 expression has been reported not only in rheumatoid arthritis (RA) (56) but also in atherosclerotic plaques (57, 58). Increased MMP-1 expression is also implicated in plaque rupture (59) and in *in vitro* endothelial cell senescence (60). A previous study demonstrated that oxLDL but not native LDL upregulated MMP-1 protein expression in human coronary endothelial cells (61). Our present study demonstrated that MMP-1 protein expression was dramatically induced in HAECs by culture medium containing NETs components with subsequent coincubation with oxLDL; whereas, notably, the coincubation of NETs components with native LDL also induced MMP-1 expression. One possible explanation is that the coincubation of NETs components and native LDL for 24 h may cause the oxidation of LDL to produce oxLDL which acts together with NETs on HAECs.

It is noteworthy that oxLDL alone does not induce NET formation but have an ability to enhance PMA-induced NET formation associated with exaggerated inflammatory responses in HAECs. Stimulation of HL-60-derived neutrophils with PMA alone induces NET formation to a certain extent. The cell shape of HAECs changed to elongated form with decreased VE-cadherin staining, induced by the culture medium of HL-60-derived neutrophils containing NETs components. This observation is consistent with that in a recent study by Meegan et al. who demonstrated that citrullinated histone H3 causes barrier dysfunction of human umbilical vein endothelial cells characterized by the disruption of cell–cell adherent junction and cytoskeleton reorganization with increased F-actin stress fibers, leading to a decrease in intercellular barrier strength and microvascular leakage as evaluated by mouse mesenteric microvessels (62). The elongation of HAECs elicited by oxLDL causes the alteration of structural and/or mechanical integrity of the endothelial barrier (63). Notably, we found that enhanced elongation in HAECs was induced by supernatant of culture medium comprising NETs from HL-60-derived neutrophils treated with either native LDL or oxLDL. This strongly indicates that accelerated NET formation induced by oxLDL and possibly native LDL-derived oxLDL generated during incubation with NETs components, may contribute to exacerbated endothelial barrier dysfunction, thereby causing vulnerability of atherosclerotic plaque.

NETs also comprise various proteases including cathepsin G, proteinase 3, MMP-9, and elastase. Cleavage of VE-cadherin mediated by elastase, one of the proteases associated with NETs, abolishes cell–cell contacts and increases cell permeability (64). Recent evidence demonstrated that cathepsin G, which is also a NETs-associating protease, mediates the degradation of apoB in native LDL to cause LDL fusion and enhanced binding of LDL to isolated human aortic proteoglycans and human atherosclerotic lesions *ex vivo*, thereby contributing to atherogenesis (65, 66). Interestingly, it is presumable that cooperative pathophysiological relevance of proteases on NETs and lipoproteins causes the progression of atherosclerosis.

The limitations of the present study include the lack of mechanistic analysis of oxLDL-mediated enhancement of NET formation including exploring receptors involved in oxLDL-mediated NET formation. It is also important to explore oxLDL-mediated NET formation under physiologically relevant stimulation, such as inflammatory cytokines (67, 68). IL-8 is a relatively weak NET inducer compared to PMA (67), and hence, human neutrophils may utilize oxLDL for enhanced NET formation under physiological stimuli, therefore, human neutrophils should be utilized to explore the contribution of oxLDL to NET formation. Future research is desired to investigate the mechanisms behind oxLDL-mediated acceleration of NET formation by human neutrophils.

In conclusion, we demonstrated in this study that oxLDL has the ability to enhance NET formation. In addition, oxLDL and NETs components have synergistic effects on morphological changes and inflammatory responses in HAECs. NET formation may possibly amplify the production of oxidatively and proteolytically modified LDL which can augment further production of oxLDL and NET formation. Our study provides information on the novel possibility that circular oxLDL and vascular NETs act together as risk factors for the initiation and progression of atherosclerosis and other vascular diseases.

## Data Availability

All datasets generated for this study are included in the manuscript and/or the Supplementary Files.

## Ethics Statement

Written informed consent in accordance with the Declaration of Helsinki was prepared and all human subjects voluntarily gave their signatures for participation in this study. The protocol was approved by the Ethical Committee of Showa University School of Pharmacy (No. 231).

## Author Contributions

TO designed the research projects. TO and HO performed the experiments and analyzed the data. TO and SI performed the isolation and oxidation of lipoproteins. TO and TT performed the electron microscopic analysis. NS, TA, and RK participated in the interpretation of the data and the discussion. HI supervised the study. TO and HI prepared the figures and wrote the manuscript. All authors reviewed the manuscript.

### Conflict of Interest Statement

The authors declare that the research was conducted in the absence of any commercial or financial relationships that could be construed as a potential conflict of interest.

## Abbreviations

AAA: abdominal aortic aneurysm
apoB: apolipoprotein B
AtRA: all-trans retinoic acid
BSA: bovine serum albumin
DAPI: 4′,6-diamidino-2-phenylindole dihydrochloride
FBS: fetal bovine serum
HAEC: human aortic endothelial cell
HRP: horseradish peroxidase
LDL: low-density lipoprotein
MMP: matrix metalloproteinase
MPO: myeloperoxidase
NETs: neutrophil extracellular traps
oxLDL: oxidatively modified low-density lipoprotein
PAD4: peptidyl arginine deiminase 4
PAGE: polyacrylamide gel electrophoresis
PBS: phosphate-buffered saline
PKC: protein kinase C
PMA: phorbol 12-myristate 13-acetate
PVDF: polyvinylidene difluoride
RA: rheumatoid arthritis
ROS: reactive oxygen species
RT: room temperature
SEM: scanning electron microscope
TBS: Tris-buffered saline
TTBS: Tris-buffered saline (TBS) containing 0. 1% Tween-20.
