# Supplementary material for: Cooperative Action of Oxidized Low-Density Lipoproteins and Neutrophils on Endothelial Inflammatory Responses Through Neutrophil Extracellular Trap Formation
Source: Front Immunol. 2019 Aug 9;10:1899. doi: 10.3389/fimmu.2019.01899 (PMC6696608; doi:10.3389/fimmu.2019.01899)
Supplement: Supplementary file 1 [file Presentation_1.pdf]

Supplementary Material

1.1 Supplementary Figures

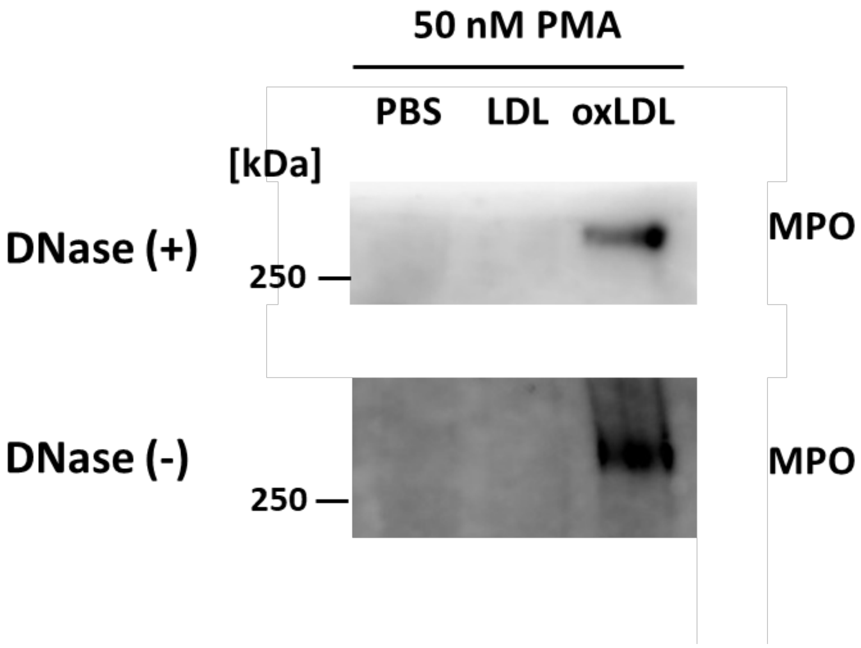

Supplemental Figure 1 Western blot analysis of MPO in culture medium of HL-60-derived neutrophils after NETs induction for 30 min followed by 20 µg/mL native LDL or oxLDL treatments for additional 2 h. Samples were treated with or without DNase I to digest DNA strands in NETs, and were subjected to 3–20 % native PAGE followed by western blot analysis.

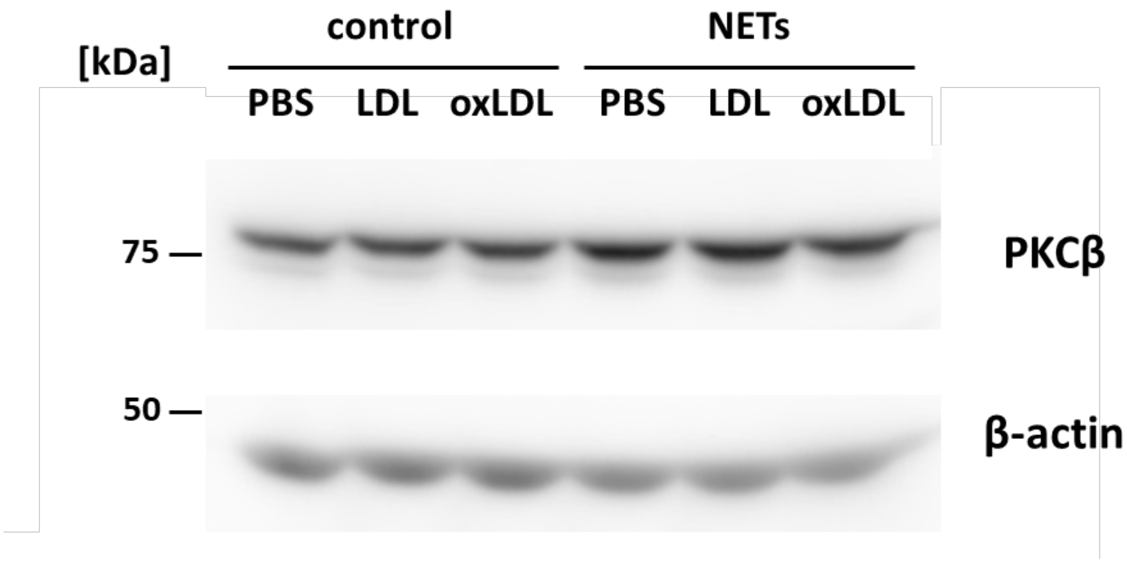

Supplemental Figure 2 Western blot analysis of PKCβ in HAECs after 24 h stimulation with culture medium from HL-60-derived neutrophils as described in the figure legend of Figure 5.

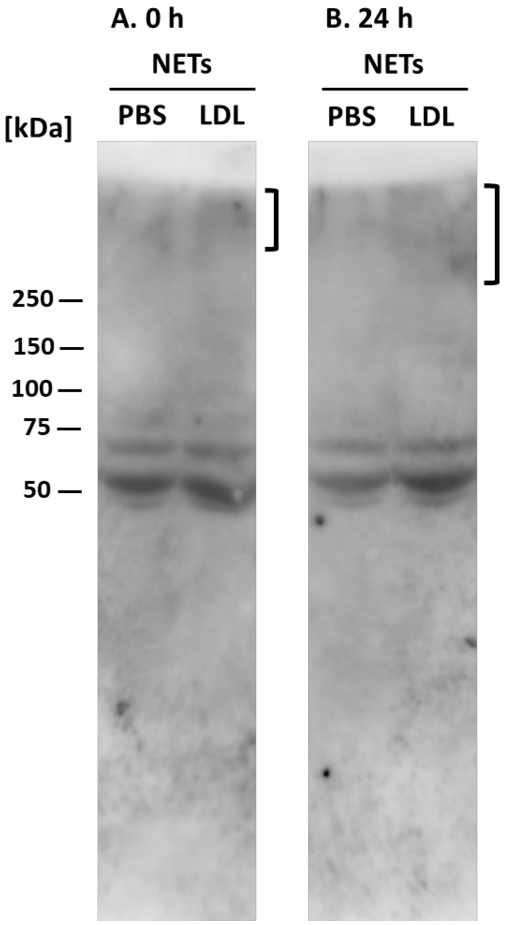

Supplemental Figure 3 Possible change in LDL during incubation with HAECs and NET components. Western blot analysis of MPO in culture medium of HAECs (A) before and (B) after stimulation for 24 h with culture medium from HL-60-derived neutrophils as described in the legend of Figure 5. The samples were subjected to 10 % SDS-PAGE (reducing condition) followed by western blot analysis. The majority of MPO was detected at 60 kDa as the monomer form. MPO was also detected above 250 kDa, indicating covalent binding with apoB in native LDL, which was increased after 24 h incubation with HAECs.
